# PM2.5 exposure aggravates oligomeric amyloid beta-induced neuronal injury and promotes NLRP3 inflammasome activation in an in vitro model of Alzheimer’s disease

**DOI:** 10.1186/s12974-018-1178-5

**Published:** 2018-05-02

**Authors:** Bian-Rong Wang, Jian-Quan Shi, Nian-Nian Ge, Zhou Ou, You-Yong Tian, Teng Jiang, Jun-Shan Zhou, Jun Xu, Ying-Dong Zhang

**Affiliations:** 1 Department of Neurology, Jiangsu Geriatric Hospital, Nanjing, 210024 Jiangsu Province People’s Republic of China; 2 Department of Neurology, Nanjing First Hospital, Nanjing Medical University, No.68, Changle Road, Nanjing, 210006 Jiangsu Province People’s Republic of China; 3 Department of Neurology, Beijing Tiantan Hospital, Capital Medical University, No. 6 Tiantanxili, Dongcheng District, Beijing, 100050 People’s Republic of China; 4 Jiangsu Key Laboratory of Integrated Traditional Chinese and Western Medicine for Prevention and Treatment of Senile Diseases, School of Medicine, Yangzhou University, Yangzhou, 225001 Jiangsu Province People’s Republic of China; 5 Department of Neurology, Northern Jiangsu People’s Hospital, Clinical Medical College of Yangzhou University, Yangzhou, 225001 Jiangsu Province People’s Republic of China

**Keywords:** Alzheimer’s disease, PM2.5, Neuronal injury, Inflammation, NLRP3 inflammasome, ROS

## Abstract

**Background:**

Numerous studies suggested that PM2.5 exposure was associated with increased risk of Alzheimer’s disease (AD). But the precise mechanisms by which PM2.5 contributed to AD pathogenesis have not been clarified.

**Methods:**

In the presence or absence of neurons, oligomeric amyloid beta (oAβ)-primed microglia were stimulated with PM2.5. Firstly, we determined the effects of PM2.5 exposure on neuronal injury and inflammation in neurons-microglia co-cultures. Then, we examined whether NLRP3 inflammasome activation was involved in PM2.5-induced inflammation. After that, we investigated whether PM2.5 exposure increased ROS level in oAβ-stimulated microglia. At last, we examined whether ROS and NLRP3 inflammasome activation was required for PM2.5-induced neuronal injury in neurons-microglia co-cultures.

**Results:**

In the present study, we showed that PM2.5 exposure aggravated oAβ-induced neuronal injury and inflammation in neurons-microglia co-cultures via increasing IL-1β production. Further, PM2.5-induced IL-1β production in oAβ-stimulated microglia was possibly dependent on NLRP3 inflammasome activation. Meanwhile, PM2.5 exposure increased ROS level in oAβ-stimulated microglia. ROS was required for PM2.5-induced IL-1β production and NLRP3 inflammasome activation in oAβ-stimulated microglia. More importantly, ROS and NLRP3 inflammasome activation was required for PM2.5-induced neuronal injury in neurons-microglia co-cultures.

**Conclusions:**

For the first time, these results suggested that the effects of PM2.5 under AD context were possibly mediated by NLRP3 inflammasome activation, which was triggered by ROS. Taken together, these findings have deepened our understanding on the role of PM2.5 in AD pathogenesis.

## Background

Alzheimer’s disease (AD) is the most common cause of dementia in the elderly. Unfortunately, the pathogenesis of AD still remains elusive [1]. There is currently an increasing interest in the association between air pollutant and AD. Air pollution is comprised of a diverse mixture of particulate matter (PM), gases, organic compounds, and metals present in outdoor and indoor air [2]. Toxic effects of environmental toxicants have been identified in in vitro and animal studies. Long-term exposures to environmental toxicants are speculated to trigger neuroinflammation and neuropathology, which paved the way for developing AD [3].

Of these environmental toxicants, PM poses severe health threats. PM is divided into three major size categories: ultra-fine PM (UFPM, < 0.1 μM), fine PM (PM2.5, < 2.5 μM), and coarse PM (PM10, < 10 and > 2.5 μM). PM2.5 are mainly composed of compounds of both organic and inorganic, including sulfates, nitrates, carbon, ammonium, hydrogen ions, lipopolysaccharides (LPS), metals, and water [4]. A population-based cohort study suggested that higher concentration of PM2.5 exposure was associated with increased risk of newly diagnosed AD [5]. But the precise mechanisms by which PM2.5 contributed to AD pathogenesis have not been clarified.

Neuroinflammation associated with microglia has been identified as a major contributor to AD pathogenesis [6]. Long-term exposure to PM2.5 has been reported to be closely associated with neuroinflammation in human [7]. Meanwhile, several lines of evidence suggested that PM2.5 exposure aggravated neuroinflammation in the brains of mice and rats [8–11]. A pilot study suggested that prolonged exposure to PM2.5 had the potential to alter the brain inflammatory phenotype and promote the development of early AD-like pathology [12]. However, the underlying mechanisms by which PM2.5 led to neuroinflammation under AD context remained largely unclear.

The NLRP3 inflammasome is a cytoplasmic multiprotein complex that regulates the cleavage of IL-1β precursors. Activation of the NLRP3 inflammasome requires two signals. The first signal leads to the synthesis of pro-IL-1β and other components of the inflammasome, such as NLRP3. The second signal results in the assembly of the NLRP3 inflammasome, caspase-1 activation, and IL-1β secretion [13]. NLRP3 inflammasome plays a pivotal role in Aβ-induced inflammation [14]. Furthermore, NLRP3 inflammasome regulates the phenotype and function of microglia, which eventually affects amyloid beta (Aβ) pathology and behavioral deficits in AD transgenic mice [15]. Hence, NLRP3 inflammasome has been regarded as therapeutic targets for AD [16]. On the other hand, several lines of evidence have indicated that particulate matter could induce NLRP3 inflammasome activation in airway epithelial cells [17, 18].

On consideration of the above evidence, we hypothesized that PM2.5 exposure aggravated oligomeric Aβ (oAβ)-induced neuronal injury and inflammation in neurons-microglia co-cultures via increasing IL-1β production, which was mediated by NLRP3 inflammasome activation. For the first time, we show that PM2.5 exposure aggravates oAβ-induced neuronal injury and inflammation in an in vitro model of AD. Meanwhile, we reveal that the aforementioned effects of PM2.5 are mediated by NLRP3 inflammasome activation. Taken together, these findings have deepened our understanding on the role of PM2.5 in AD pathogenesis.

## Methods

### Reagents

PM2.5 was purchased from the National Institute for Standards and Technology (Gaithersburg, MD, USA). Aβ1-42, LPS, NADPH, lucigenin, diphenylene iodonium (DPI, NADPH oxidase inhibitor), and *N*-acetyl-l-cysteine (NAC) were purchased from Sigma-Aldrich (St. Louis, MO, USA). Z-VAD-FMK (pan-caspase inhibitor) and Z-YVAD-FMK (caspase-1 inhibitor) were purchased from Calbiochem (Gibbstown, NJ, USA). IL-1 receptor antagonist (IL-1ra) was purchased from R&D (Minneapolis, MN, USA). MitoQ was purchased from Focus Biomolecules (Plymouth Meeting, PA, USA).

### Cell culture

Pregnant C57BL/6 mice were provided by Animal Experiment Center of Nanjing First Hospital, Nanjing Medical University. All animal procedures were carried out in accordance with Chinese Association for Laboratory Animal Sciences Guide for Care and Use of Laboratory Animals and were approved by the Nanjing Medical University Experimental Animal Care and Use Committee.

Primary microglial cells were prepared from the brains of C57BL/6 at postnatal day 1 as previously described [19]. Briefly, the leptomeningeal and meninges blood vessels were removed from the cortex. Cells were dissociated by trituration and cultured in DMEM containing 10% heat-inactivated fetal bovine serum, 2 mM L-glutamine and 1% penicillin/streptomycin (Hyclone, Logan, UT, USA) for 2–3 weeks in poly-d-lysine-coated 75-cm^2^ flasks to form a confluent glial monolayer. Half of the medium was replaced with fresh cell culture medium every 3 days. To collect microglial cells, the cultures were shaken on a rotary shaker (placed in a cell culture incubator, 37 °C and 5% CO2) at 250 rpm for 3 h. The detached microglial cells were collected by centrifugation, and the enriched microglial cell suspension was plated onto poly-d-lysine-coated 6-well plates containing poly-d-lysine-coated glass coverslips. After the cells are attached, the medium was replaced with fresh culture medium. The purity of the isolated microglia was determined by immunostaining with antibodies against Iba1. In average, over 90% of cultured cells were immunostained with Iba1.

Primary neurons were prepared from the cortices of mouse embryos at embryonic day 17 (E17) as previously described [20]. Briefly, cortical fragments were dissociated into single cells and resuspended in neurobasal medium supplemented with 2% B27 and 1% penicillin/streptomycin. Half of the medium was replaced with fresh cell culture medium every 3 days. The purity of the culture was more than 95% as determined by NeuN-specific immunostaining (Merck Millipore, Billerica, MA, USA).

Neurons-microglia co-cultures were performed as previously described [21, 22]. Microglia and neurons were separately seeded into 6-well transwell plates (0.4 μm pore size, Corning Costar, St Louis, MO, USA). Neurons in neuronal medium (neurobasal medium supplemented with 2% B27 and 1% penicillin/streptomycin) were plated in the lower chamber. Before co-culture, microglia were activated with LPS (1 μg/ml) for 3 h (LPS priming). Inserts containing microglia were washed with fresh serum-free DMEM and then plated in the upper chamber. Then, neurons-microglia co-cultures were treated with or without oAβ (5 μM). After 12 h, neurons-microglia co-cultures were treated with or without PM2.5 (50 μg/ml) for 4 h.

### Preparation of oAβ

oAβ was prepared as previously described [21, 22]. Briefly, oAβ1-42 was prepared by dissolving Aβ1-42 to 1 mmol/l in 100% 1,1,1,3,3,3-hexafluoro-2-propanol. 1,1,1,3,3,3-Hexafluoro-2-propanol was dried by a vacuum desiccator and resuspended to 5 mmol/l in DMSO. To form oligomers, the amyloid peptide was diluted to a final concentration of 100 mmol/l with Ham’s F-12, incubated at 4 °C for 24 h and then immediately added to cultures at a final concentration 5 μmol/l. Formation of oAβ was confirmed by western blotting as previously described [21, 22].

### Flow cytometry for cell apoptosis, intracellular reactive oxygen species level, and mitochondrial ROS level

To evaluate apoptosis, annexin V/propidium iodide (PI) analyses were performed as previously described [23]. Briefly, 5 × 10^5^ cells were collected by centrifugation and resuspended in 500 μl binding buffer. Then, 5 μl annexin V and 10 μl PI were added. After incubation at room temperature for 5 min in the dark, cells were analyzed by flow cytometry. Data were analyzed using CellQuest Pro software (BD).

The level of intracellular ROS was measured by DCFH oxidation using a commercial detection kit (Beyotime Biotechnology, Shanghai, China), as previously reported [24]. Briefly, 10 mM (0.1 ml) DCFH-DA was diluted 1000-fold in DMEM without serum, to give a 10 μM concentration of DCFH-DA. The treated cells were washed three times with PBS and then incubated in 10 mM DCFH-DA for 30 min at 37 °C without light. After being washed three times with PBS, cells were analyzed by flow cytometry. Data were analyzed using CellQuest Pro software (BD).

The level of mitochondrial ROS was measured by MitoSOX Red superoxide indicator staining (Invitrogen, Carlsbad, CA, USA), as previously reported [22]. Briefly, 50 μg MitoSOX Red superoxide indicator was dissolved in 13 μl DMSO to make 5 mM MitoSOX reagent stock solution. Then, the stock solution was diluted 1000-fold in buffer to make a 5 μM MitoSOX reagent working solution. Microglia seeded (5 × 10^5^) in 24-well plates were exposed to 1 ml MitoSOX reagent working solution and then incubated for 10 min at 37 °C. Cells were collected and washed twice by buffer, and cells were analyzed by flow cytometry. Data were analyzed using CellQuest Pro software (BD).

### MTT assay

The MTT assay was performed using a Cell Counting Kit (Beyotime Biotechnology, Shanghai, China) as described previously [23]. Briefly, neurons were seeded at a density of 2 × 10^5^ cells/cm^2^ and received different treatments. Then, 10-μl CCK-8 solution was added to each well. Absorbance at 450 nm was measured 2 h after incubation in the dark.

### In vitro validation of oxidative capacity

In vitro validation of oxidative capacity was performed as described previously [17]. Primary microglial cells were seeded at a density of 5 × 10^4^ cells per dish. Cells were washed three times with PBS and incubated with a cell-permeable redox-sensitive fluorescent dye (CellROX, Invitrogen), followed by washes with PBS. Live-cell confocal imaging was performed within 30 min of washes. Signal intensity per unit area was determined for three fields of view from three different samples.

### Enzyme-linked immunosorbent assay

Mouse IL-1β ELISA Kits were obtained from Invitrogen (Carlsbad, CA, USA). ELISA was performed according to manufacturer’s instruction.

### Western blotting

The following primary antibodies were used: mouse anti-Caspase-1 (1:200; Santa Cruz Biotechnology, Santa Cruz, CA, USA), rabbit anti-NLRP3 antibody (1:800; Santa Cruz Biotechnology, Santa Cruz, CA, USA), and mouse anti-β-actin antibody (1:1000; Santa Cruz Biotechnology, Santa Cruz, CA, USA).

Western blotting was performed as described elsewhere [25]. Cells were lysed in radio-immunoprecipitation assay (RIPA) lysis buffer supplemented with protease inhibitors (Complete; Roche, Indianapolis, IN, USA). The lysates were resolved by SDS-PAGE. Supernatants and the final pellets from each sample were heat-blocked for 5 min in loading buffer (125 mM Tris-HCl, 20% glycerol, 10% 2-mercaptoethanol, 4% SDS, 0.02% bromophenol blue, pH 6.8) and then subjected to electrophoresis on 10–20% Tris-glycine SDS-PAGE gels. Proteins were then electrically transferred to a transfer membrane (BioRad, Hercules, CA, USA) and blocked for 1 h in Tris-HCl-buffered saline containing 5% skim milk and 0.1% Tween. The membranes were incubated in primary antibodies at 4 °C overnight in TBS buffer containing 5% bovine albumin. The membranes were then rinsed with TBS buffer containing 0.1% Tween 20, incubated with horseradish peroxidase (HRP)-labeled secondary antibody, and then stained with detection reagents. Finally, the membranes were developed using the enhanced chemiluminescence (ECL) system. Immunoreactivity was quantified using ImageJ software (Rasband, MD, USA).

### Measurement of caspase-1 activity

Microglia cells, seeded at a density of 1 × 10^7^ cells and treated as described, were measured for caspase-1 activity according to the manufacturer’s instruction (Beyotime Biotechnology, Shanghai, China).

### Measurement of NADPH oxidase activity

NADPH oxidase activity was measured by lucigenin-enhanced chemiluminescence, as previously reported [26]. Microglial cells were collected, and total protein concentration was determined using a bicinchoninic acid protein assay kit (Beyotime Biotechnology, Shanghai, China). One hundred micromolars of NADPH and 5 μM dark-adapted lucigenin were added into 0.5-ml microcentrifuge tubes just before reading. Light emission was recorded over 5 min, and values were expressed as mean light units per minute per milligram of protein. Using this method, the superoxide anion production also represents NADPH oxidase activity.

### Statistical analysis

Statistical analysis was performed using SPSS software (version 17.0, SPSS, Chicago, IL, USA). All data are expressed as mean ± SEM. Statistical significance was assessed by one-way ANOVA followed by the LSD procedure. If variances were unequal, the Games-Howell procedure was followed by ANOVA. The level of statistical significance was defined as *P* < 0.05.

## Results

### PM2.5 exposure aggravates oAβ-induced neuronal injury and inflammation in neurons-microglia co-cultures via increasing IL-1β production

**Fig. 1:**
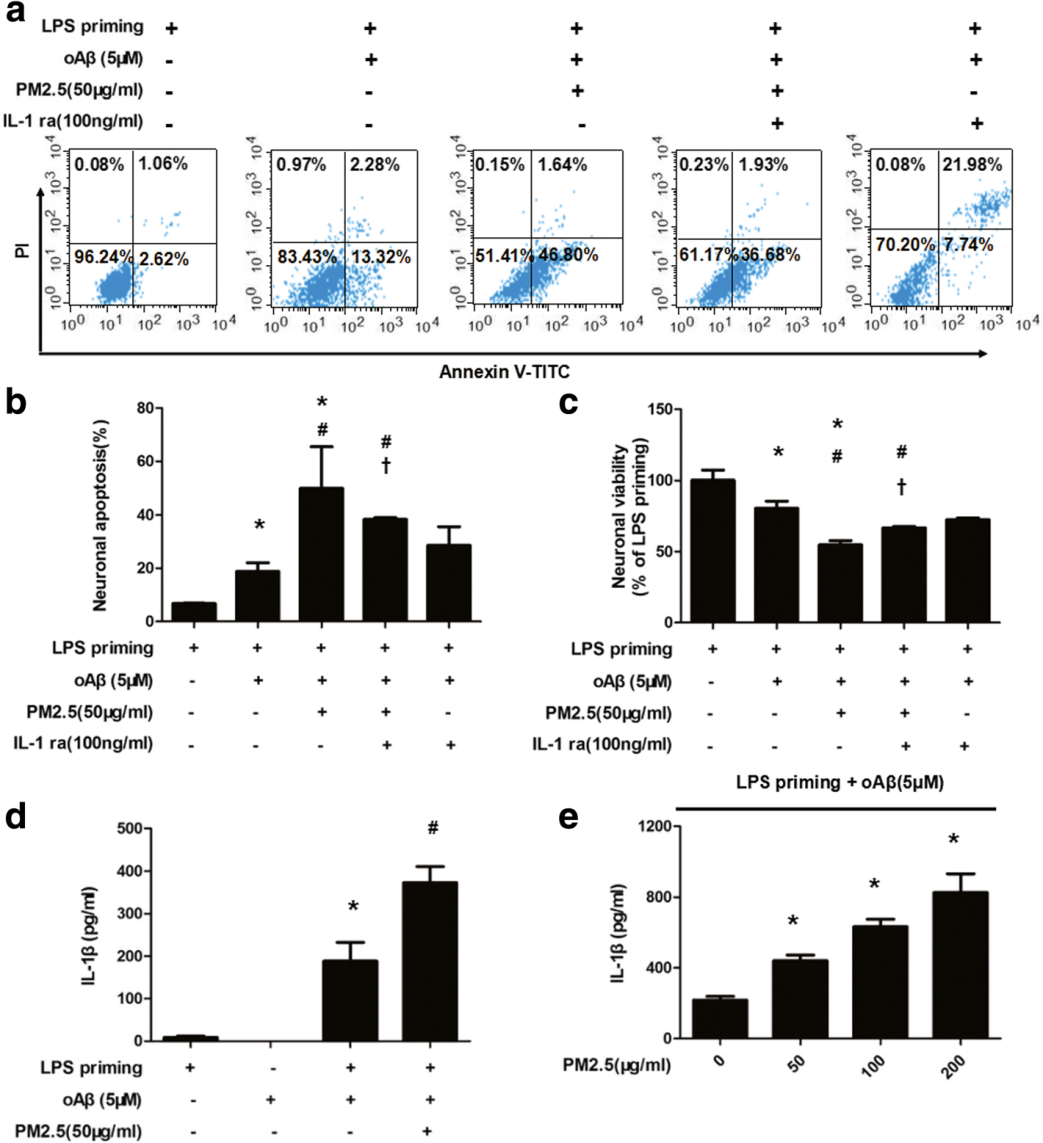
PM2.5 exposure aggravates oAβ-induced neuronal injury and inflammation in neurons-microglia co-cultures via increasing IL-1β production. In transwell co-culture system, neurons in the neuronal medium were plated in the lower chamber. Before co-culture, microglia were activated with LPS (1 μg/ml) for 3 h (LPS priming). Inserts containing microglia were washed with fresh serum-free DMEM and then plated in the upper chamber. Then, neurons-microglia co-cultures were treated with or without oAβ (5 μM). After 12 h, neurons-microglia co-cultures were treated with or without PM2.5 (50 μg/ml) for 4 h. **a**, **b** Apoptosis of co-cultured neurons was evaluated by flow cytometry with annexin V/PI staining. **c** Cell viability of co-cultured neurons was assessed via MTT assay. *P < 0.05 vs. neurons in co-cultures with LPS-primed microglia. ^#^*P* < 0.05 vs. neurons in co-cultures with oAβ-stimulated microglia. ^†^*P* < 0.05 vs. neurons in co-cultures with PM2.5-oAβ-stimulated microglia. **d** Microglia were activated with LPS (1 μg/ml) for 3 h (LPS priming), and then oAβ (5 μM) was added into the culture medium. After 12 h, the cells were stimulated with PM2.5 (50 μg/ml) for 4 h. The concentration of IL-1β in the culture supernatant was then measured by ELISA. **e** The cells were stimulated with PM2.5 (0, 50, 100, 200 μg/ml) for 4 h. The concentration of IL-1β in the culture supernatant was measured by ELISA. All figures are representative of three independent experiments, performed in triplicate. *P < 0.05 vs. LPS-primed microglia. ^#^*P* < 0.05 vs. LPS-primed microglia stimulated with oAβ

First, we determined the effects of PM2.5 exposure on neuronal injury in neurons-microglia co-cultures by using transwell co-culture system. Neurons in the neuronal medium were plated in the lower chamber. Before co-culture, microglia were activated with LPS (1 μg/ml) for 3 h (LPS priming). Inserts containing microglia were washed with fresh serum-free DMEM and then plated in the upper chamber. Then, neurons-microglia co-cultures were treated with or without oAβ (5 μM). After 12 h, neurons-microglia co-cultures were treated with or without PM2.5 (50 μg/ml) for 4 h. We measured neuronal apoptosis using flow cytometry with annexin V/PI staining. When compared with neurons in co-cultures with LPS-priming microglia, oAβ stimulation and oAβ-PM2.5 dual stimulation increased neuronal apoptosis (oAβ stimulation: 18.86 vs. 6.77%, *P* < 0.05; oAβ-PM2.5 dual stimulation: 50.02 vs. 6.77%, *P* < 0.05; Fig. 1a, b). When compared with neurons in co-cultures with oAβ-stimulated microglia, PM2.5 exposure increased neuronal apoptosis (50.02 vs. 18.86%, *P* < 0.05; Fig. 1a, b). To validate the association between PM2.5 and neuronal viability, we then detected neuronal viability via MTT assay. As expected, when compared with neurons in co-cultures with LPS-priming microglia, oAβ stimulation and oAβ-PM2.5 dual stimulation decreased neuronal viability by 19.42% (*P* < 0.05) and 45.14% (*P* < 0.05) (Fig. 1c). When compared with neurons in co-cultures with oAβ-stimulated microglia, PM2.5 exposure decreased neuronal viability by 25.72% (Fig. 1c).

Then, we assessed whether PM2.5 induced inflammation in oAβ-stimulated microglia. Microglia were activated with LPS (1 μg/ml) for 3 h (LPS priming), and then oAβ (5 μM) was added into the culture medium. After 12 h, the cells were stimulated with PM2.5 (50 μg/ml) for 4 h. The concentration of IL-1β in the culture supernatant was then measured by ELISA. As shown in Fig. 1d, PM2.5 exposure increased IL-1β concentration by 97.80% in oAβ-stimulated microglia. Meanwhile, oAβ alone did not induce IL-1β production as previously described (Fig. 1d). Further, the effects of PM2.5 exposure on IL-1β production were dose-dependent (Fig. 1e). We also assessed whether IL-1 receptor antagonist (IL-1ra) affected the apoptosis or viability of the co-cultured neurons. In transwell co-culture system, PM2.5-oAβ-stimulated neurons-microglia co-cultures were treated with or without IL-1ra. IL-1ra reduced neuronal apoptosis by 11.62% (Fig. 1a, b). Meanwhile, IL-1ra increased neuronal viability by 11.90% (Fig. 1c). Taken together, these results indicated that PM2.5 exposure aggravated oAβ-induced neuronal injury and inflammation in neurons-microglia co-cultures via increasing IL-1β production.

### PM2.5-induced IL-1β production in oAβ-stimulated microglia is possibly dependent on NLRP3 inflammasome activation

**Fig. 2:**
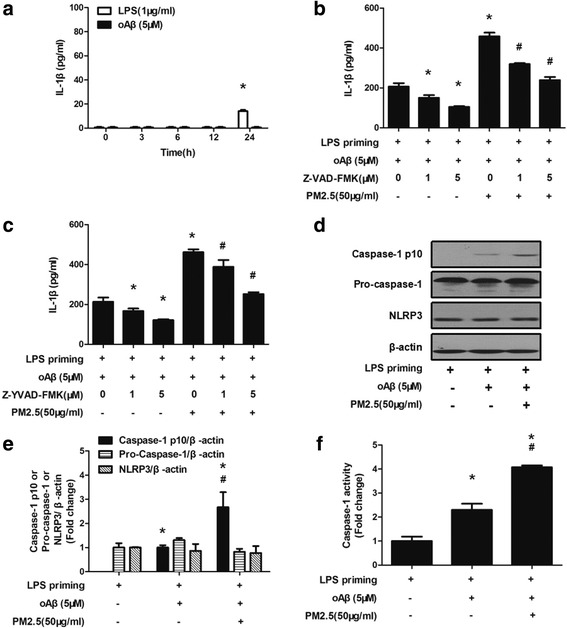
PM2.5-induced IL-1β production in oAβ-stimulated microglia is possibly dependent on NLRP3 inflammasome activation. Microglia were stimulated by LPS or oAβ alone for varying time, and the concentration of IL-1β in the culture supernatant was measured by ELISA (**a**). Microglia primed with LPS for 3 h were washed with fresh serum-free DMEM. LPS-primed microglia were stimulated with oAβ for 12 h and treated with pan-caspase inhibitor Z-VAD-FMK (**b**) or caspase-1 inhibitor Z-YVAD-FMK (**c**) for 30 min before PM2.5 exposure. After PM2.5 exposure for 4 h, IL-1β concentration in the culture supernatant was measured by ELISA. All figures are representative of three independent experiments, performed in triplicate. *P < 0.05 vs. LPS-primed microglia stimulated with oAβ. ^#^*P* < 0.05 vs. LPS-primed microglia stimulated with oAβ and PM2.5. **d**, **e** LPS-primed microglia were stimulated with oAβ for 12 h and then treated with PM2.5 for 4 h. The protein levels of Caspase-1 p10, pro-caspase-1, and NLRP3 were assessed by western blotting. β-actin was used as loading control. **f** LPS-primed microglia were stimulated with oAβ for 12 h and then treated with PM2.5 for 4 h. Caspase-1 activity was measured. All figures are representative of three independent experiments, performed in triplicate. *P < 0.05 vs. LPS-primed microglia. ^#^*P* < 0.05 vs. LPS-primed microglia stimulated with oAβ

We next examined whether NLRP3 inflammasome activation was involved in the PM2.5-induced IL-1β production. We first assessed whether LPS or oAβ alone induced IL-1β production in microglia. Microglia were stimulated by LPS or oAβ alone for varying time, and the concentration of IL-1β in the culture supernatant was measured by ELISA. We found that LPS stimulation alone for 0–12 h failed to induce IL-1β production. We detected IL-1β production when microglia were stimulated by LPS alone for 24 h (Fig. 2a). Meanwhile, we found that oAβ stimulation alone for 0–24 h failed to induce IL-1β production (Fig. 2a). Then, LPS-primed microglia were stimulated with oAβ for 12 h and treated with pan-caspase inhibitor Z-VAD-FMK or caspase-1 inhibitor Z-YVAD-FMK for 30 min before PM2.5 exposure. After PM2.5 exposure for 4 h, IL-1β concentration in the culture supernatant was measured. Both Z-VAD-FMK and Z-YVAD-FMK dose-dependently decreased IL-1β production in the culture supernatant (Fig. 2b, c). Next, we assessed the cleaved fraction of caspase-1 (Casp-1 p10), pro-caspase-1, and NLRP3 by western blotting. When compared with LPS-primed microglia, both oAβ stimulation and PM2.5-oAβ dual stimulation induced the cleavage of caspase-1 and generated caspase-1 p10 subunit (Fig. 2d, e). When compared with the oAβ-stimulated microglia, PM2.5 exposure increased the generation of caspase-1 p10 subunit by nearly 2.76-fold (*P* < 0.05) (Fig. 2d, e). We did not observe significant changes in pro-caspase-1 or NLRP3 protein expression after oAβ stimulation or PM2.5-oAβ dual stimulation (Fig. 2d, e). To further the effects of PM2.5 exposure on NLRP3 inflammasome activation, we detected caspase-1 activity in microglia. When compared with the LPS-primed microglia, both oAβ stimulation and PM2.5-oAβ dual stimulation increased caspase-1 activity by nearly 2.30-fold (*P* < 0.05) and 4.07-fold (*P* < 0.05), respectively (Fig. 2f). When compared with the oAβ-stimulated microglia, PM2.5 exposure increased caspase-1 activity by nearly 1.77-fold (*P* < 0.05) (Fig. 2f). Taken together, these results suggested that PM2.5-induced IL-1β production in oAβ-stimulated microglia is possibly dependent on NLRP3 inflammasome activation.

### PM2.5 exposure increases ROS level in oAβ-stimulated microglia

**Fig. 3:**
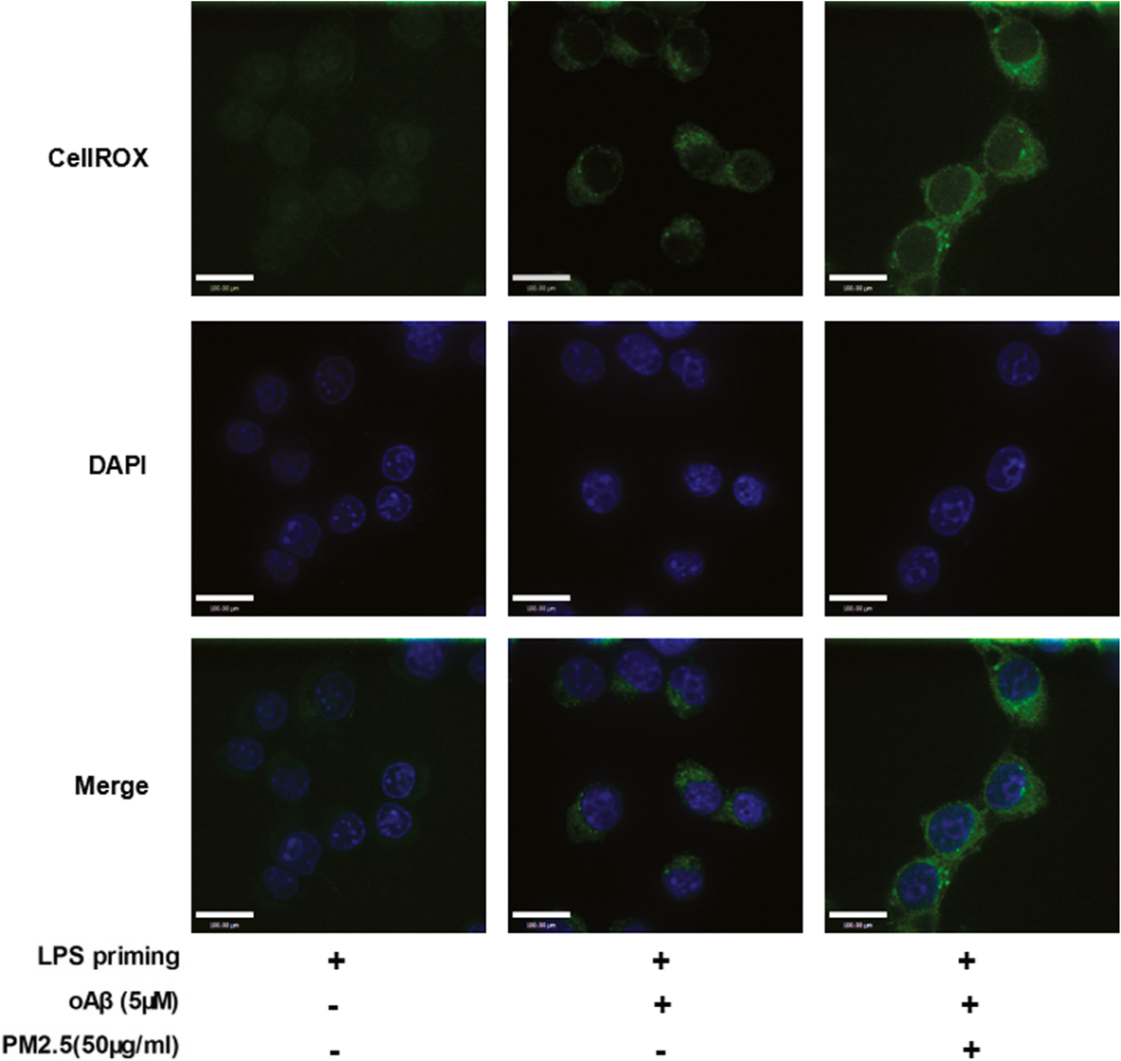
PM2.5 exposure increases ROS level in oAβ-stimulated microglia. Microglia with different treatment were incubated with CellROX (green). Meanwhile, cells were co-stained with DAPI (blue). Staining images of microglia with different treatments were shown. Scale bar = 20 μm

**Fig. 4:**
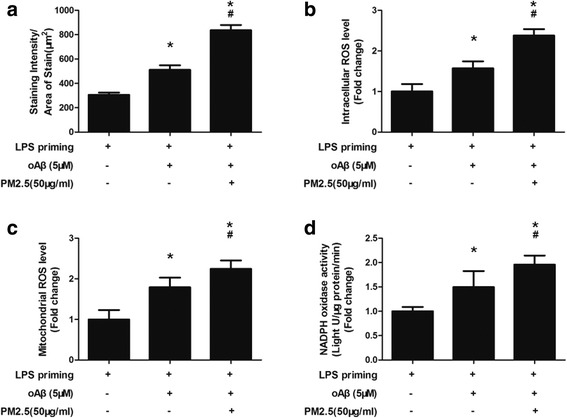
PM2.5 exposure increases ROS level in oAβ-stimulated microglia. Microglia with different treatment were incubated with CellROX. **a** Average staining intensity per unit area of stain quantified for three random fields of view. Microglia were treated with different stimulation, according to different groups. **b** The degree of oxidative stress (indicated by intracellular ROS level) was detected using a commercial detection kit. **c** The mitochondrial ROS level was detected by flow cytometry using MitoSOX Red superoxide indicator. **d** NADPH oxidase activity was tested by lucigenin-enhanced chemiluminescence. All figures are representative of three independent experiments, performed in triplicate. *P < 0.05 vs. LPS-primed microglia. ^#^*P* < 0.05 vs. LPS-primed microglia stimulated with oAβ

PM2.5 is reported to trigger oxidative stress, which is regarded as a danger signal for NLRP3 inflammasome activation. By using a cell-permeable redox-sensitive fluorescent dye (CellROX), we demonstrated that PM2.5 exposure increased staining intensity/unit area in oAβ-stimulated microglia (*P* < 0.05, Figs. 3 and 4a). To validate the association between PM2.5 and oxidative stress, the degree of oxidative stress (indicated by intracellular ROS level) was detected using a commercial detection kit. When compared with LPS-primed microglia, both oAβ stimulation and PM2.5-oAβ dual stimulation increased intracellular ROS level by nearly 1.57-fold (*P* < 0.05) and 2.38-fold (*P* < 0.05), respectively (Fig. 4b). When compared with oAβ-stimulated microglia, PM2.5 exposure increased intracellular ROS level by nearly 1.52-fold (*P* < 0.05) (Fig. 4b). In order to identify the source of ROS, we detected mitochondrial ROS level using MitoSOX Red superoxide indicator by flow cytometry. When compared with LPS-primed microglia, both oAβ stimulation and PM2.5-oAβ dual stimulation increased mitochondrial ROS level by nearly 1.79-fold (*P* < 0.05) and 2.39-fold, respectively (Fig. 4c). When compared with oAβ-stimulated microglia, PM2.5 exposure increased mitochondrial ROS level by nearly 1.34-fold (*P* < 0.05) (Fig. 4c). Meanwhile, NADPH oxidase activity was tested by lucigenin-enhanced chemiluminescence. When compared with LPS-primed microglia, both oAβ stimulation and PM2.5-oAβ dual stimulation increased NADPH oxidase activity by nearly 1.50-fold (*P* < 0.05) and 1.96-fold, respectively (Fig. 4d). When compared with oAβ-stimulated microglia, PM2.5 exposure increased mitochondrial ROS level by nearly 1.31-fold (*P* < 0.05) (Fig. 4d). Taken together, these results suggested that PM2.5 exposure increased ROS level in oAβ-stimulated microglia via both mitochondrial-mediated manner and NADPH oxidase-mediated manner.

### ROS is required for PM2.5-induced IL-1β production and NLRP3 inflammasome activation in oAβ-stimulated microglia

**Fig. 5:**
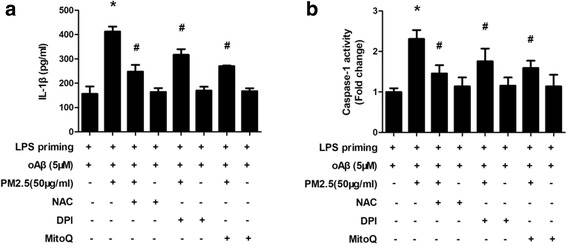
ROS is required for PM2.5-induced IL-1β production and NLRP3 inflammasome activation in oAβ-stimulated microglia. LPS-primed microglia were stimulated with oAβ for 12 h. Then, microglia were treated with ROS scavenger NAC, NADPH oxidase inhibitor DPI, and mitochondria-targeted antioxidant MitoQ for 30 min before PM2.5 exposure for 4 h. **a** IL-1β concentration in the culture supernatant was measured by ELISA. **b** Caspase-1 activity was measured. All figures are representative of three independent experiments, performed in triplicate. *P < 0.05 vs. LPS-primed microglia stimulated with oAβ. ^#^*P* < 0.05 vs. LPS-primed microglia stimulated with oAβ and PM2.5

Next, we investigated the role of ROS in IL-1β production and NLRP3 inflammasome activation in PM2.5-oAβ-stimulated microglia. LPS-primed microglia were stimulated with oAβ for 12 h. Then, microglia were treated with ROS scavenger NAC, NADPH oxidase inhibitor DPI, and mitochondria-targeted antioxidant MitoQ for 30 min before PM2.5 exposure. After PM2.5 exposure for 4 h, IL-1β concentration and caspase-1 activity in the culture supernatant were measured. NAC, DPI, and MitoQ reduced PM2.5-induced IL-1β production in oAβ-stimulated microglia by 66.30% (*P* < 0.05), 30.20% (*P* < 0.05), and 52.59% (*P* < 0.05), respectively (Fig. 5a). Further, NAC, DPI, and MitoQ decreased PM2.5-induced caspase-1 activity in oAβ-stimulated microglia by 58.59% (*P* < 0.05), 31.42% (*P* < 0.05), and 45.04% (*P* < 0.05), respectively (Fig. 5b). Taken together, these results suggested that ROS was required for PM2.5-induced IL-1β production and NLRP3 inflammasome activation in oAβ-stimulated microglia.

### ROS and NLRP3 inflammasome activation is required for PM2.5-induced neuronal injury in neurons-microglia co-cultures

**Fig. 6:**
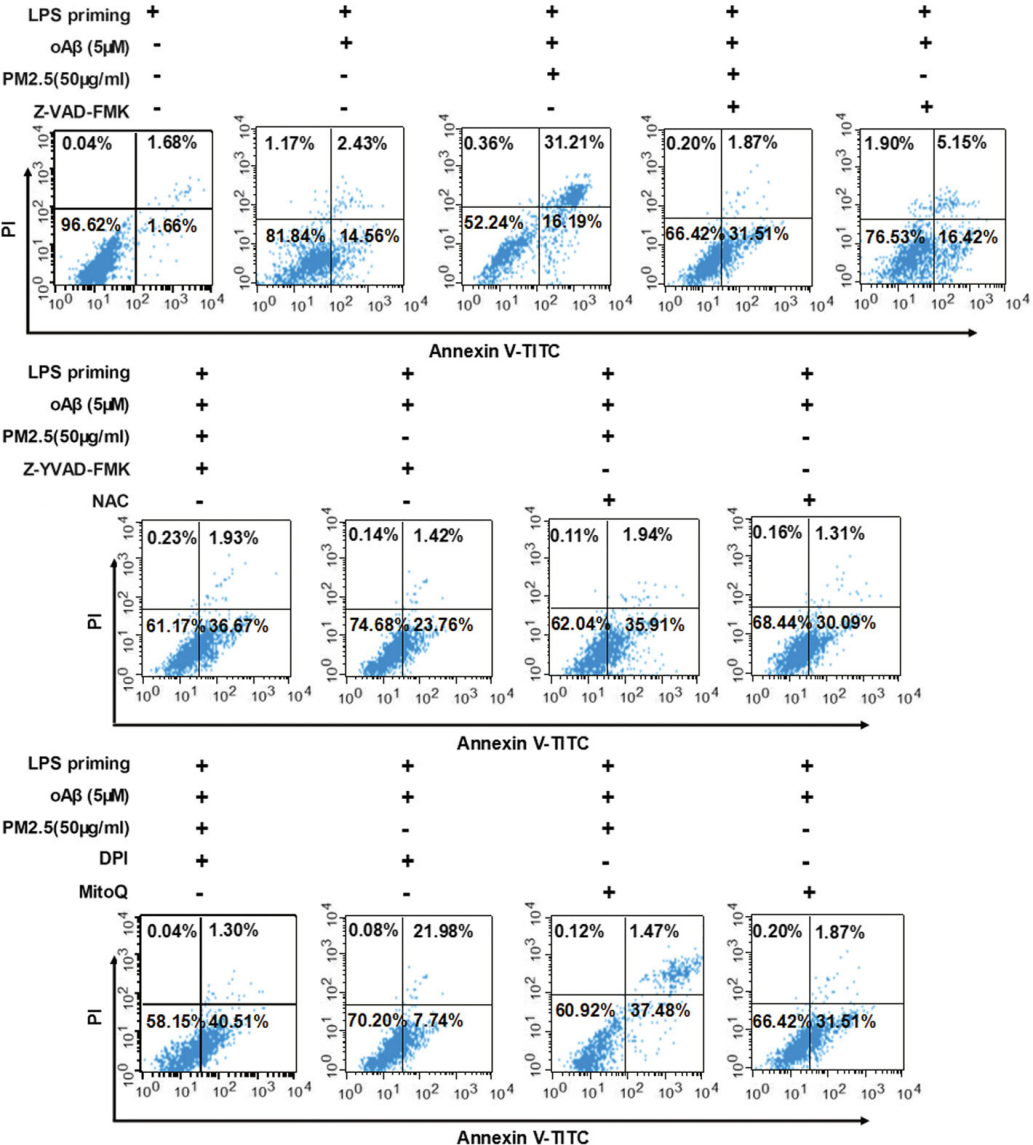
ROS and NLRP3 inflammasome activation is required for PM2.5-induced neuronal injury in neurons-microglia co-cultures. In a transwell co-culture system, neurons-microglia co-cultures were stimulated with oAβ for 12 h and treated with ROS inhibitors and caspase-1 inhibitors for 30 min before PM2.5 exposure. Z-VAD-FMK, Z-YVAD-FMK, NAC, DPI, and MitoQ were included. After PM2.5 exposure for 4 h, cell apoptosis was assessed by annexin V/PI method, and a representative experiment is shown

**Fig. 7:**
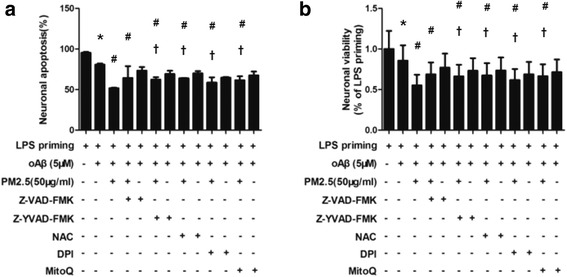
ROS and NLRP3 inflammasome activation is required for PM2.5-induced neuronal injury in neurons-microglia co-cultures. In a transwell co-culture system, neurons-microglia co-cultures were stimulated with oAβ for 12 h. Then, the co-cultures were treated with ROS inhibitors and caspase-1 inhibitors for 30 min before PM2.5 exposure for 4 h. Z-VAD-FMK, Z-YVAD-FMK, NAC, DPI, and MitoQ were included. **a** Apoptosis of co-cultured neurons were evaluated by flow cytometry with annexin V/PI staining. **b** Cell viability of co-cultured neurons were assessed via MTT assay. All figures are representative of three independent experiments, performed in triplicate. *P < 0.05 vs. neurons in co-cultures with LPS-primed microglia. ^#^*P* < 0.05 vs. neurons in co-cultures with oAβ-stimulated microglia. ^†^*P* < 0.05 vs. neurons in co-cultures with PM2.5-oAβ-stimulated microglia

Further, we investigated the effects of ROS inhibitors and caspase-1 inhibitors on neuronal injury in neurons-microglia co-cultures. In transwell co-culture system, neurons-microglia co-cultures were stimulated with oAβ for 12 h. Then, the co-cultures were treated with ROS inhibitors and caspase-1 inhibitors for 30 min before PM2.5 exposure. Z-VAD-FMK, Z-YVAD-FMK, NAC, DPI, and MitoQ were included. After PM2.5 exposure for 4 h, apoptosis and viability of co-cultured neurons were detected. Z-VAD-FMK, Z-YVAD-FMK, NAC, DPI, and MitoQ reduced neuronal apoptosis by 12.48, 10.47, 12.10, 6.80, and 9.69%, respectively (Figs. 6 and 7a). Meanwhile, Z-VAD-FMK, Z-YVAD-FMK, NAC, DPI, and MitoQ increased neuronal viability by 13.92, 11.40, 12.42, 6.87, and 11.55%, respectively (Fig. 7b). Taken together, these results suggested that ROS and NLRP3 inflammasome activation was required for PM2.5-induced neuronal injury in neurons-microglia co-cultures.

## Discussion

Firstly, we chose oAβ-treated primary neurons-microglia co-cultures as an in vitro model of AD as previously reported [21, 22]. In the present study, we showed that PM2.5 exposure aggravated the oAβ-induced neuronal injury in neurons-microglia co-cultures, as flow cytometry suggested that PM2.5 exposure increased neuronal apoptosis. A similar result was obtained by MTT assay, which revealed a corresponding decrease in neuronal viability following PM2.5 exposure. These results were in accordance with previous studies showing that PM2.5 led to neuronal injury [27, 28]. Then, we showed that PM2.5 exposure increased IL-1β production in oAβ-stimulated microglia. The result was supported by previous studies showing that PM2.5 caused IL-1β production in other cells, such as human airway epithelial cells, macrophage, ventricular myocytes, and a human keratinocyte cell line [17, 29–31]. More importantly, the effects of PM2.5 on the neuronal injury were mitigated by IL-1ra, as IL-1ra decreased neuronal apoptosis in flow cytometry and increased neuronal viability in MTT assay. The result was in agreement with a recent study from Dong et al. which showed that IL-1β-induced inflammation triggered neuronal apoptosis [32]. To our knowledge, this is the first study showing that PM2.5 exposure aggravated neuronal injury and inflammation via increasing IL-1β production under AD context.

However, the underlying mechanisms by which PM2.5 increased IL-1β production in microglia are still not fully understood. IL-1β has been regarded as a biochemical marker of NLRP3 inflammasome activation [33]. In the subsequent experiment, we found that LPS stimulation alone for 0–12 h failed to induce IL-1β production. Meanwhile, we found that oAβ stimulation alone for 0–24 h failed to induce IL-1β production. This finding was in accordance with the previous studies [22]. In general, a two-signal model has been proposed for NLRP3 inflammasome activation. The first signal (priming) is provided by microbial or endogenous molecules that induce NLRP3 and pro-IL-1β expression through activation of NF-κB; the second signal (activation) is triggered by ATP, pore-forming toxins, viral RNA, and particulate matter [34]. However, LPS is able to induce IL1β secretion and activation of inflammasome through non-canonical NLRP3 inflammasome activation and alternative NLRP3 inflammasome activation. In most studies, at least 6 h of LPS stimulation was needed to induce IL-1β secretion and activation of inflammasome in different cells [22, 35–37]. In the present study, 3 h of LPS stimulation was too short to induce IL-1β secretion and activation of inflammasome.

Then, we investigated whether NLRP3 inflammasome activation was involved in the PM2.5-induced IL-1β production. By using inhibitor, we found that both pan-caspase inhibitor Z-VAD-FMK and caspase-1 inhibitor Z-YVAD-FMK dose-dependently decreased PM2.5-induced IL-1β production in oAβ-stimulated microglia. More importantly, we observed a significant change in Casp-1 p10 protein expression in oAβ-stimulated microglia after PM2.5 exposure. Meanwhile, PM2.5 exposure increased caspase-1 activity in oAβ-stimulated microglia. Based on the above evidence, we concluded that PM2.5-induced IL-1β production in oAβ-stimulated microglia was possibly dependent on NLRP3 inflammasome activation. This finding was in accordance with the previous studies conducted in human airway epithelial cells, macrophage, ventricular myocytes, and a human keratinocyte cell line [17, 29–31]. However, gene knockout experiments have not been performed in the present study. Without data of knockout experiments, no direct evidence shows that the effects of PM2.5 were mediated by NLRP3 inflammasome activation. On the other hand, further research is needed to investigate the role and mechanism of other cytokines in the effects of PM2.5.

Increasing number of studies link oxidative stress to NLRP3 inflammasome activation in a wide range of autoinflammatory and autoimmune disorders [38]. Moreover, PM2.5 is demonstrated to trigger oxidative stress in some diseases, including cardiovascular diseases, respiratory diseases, diabetes mellitus, and Parkinson’s disease [39–42]. As expected, in the present study, we showed that PM2.5 exposure increased ROS level in oAβ-stimulated microglia, as intracellular ROS level was significantly increased following PM2.5 exposure. The enhancement in oxidative stress seemed to be mediated by the activation of NADPH oxidase and mitochondria damage. This finding was supported by previous reports showing that PM2.5 induced oxidative stress via both mitochondrial-mediated manner and NADPH oxidase-mediated manner in human bronchial epithelial cells and human myeloid leukemia cells [43, 44].

Lastly, ROS inhibitors (NAC, DPI, and MitoQ) were used to investigate the role of ROS in IL-1β production and NLRP3 inflammasome activation. We found that all the ROS inhibitors decreased IL-1β production and caspase-1 activity in PM2.5-oAβ-stimulated microglia. Meanwhile, we investigated the effects of ROS inhibitors and caspase-1 inhibitors on neuronal injury in neurons-microglia co-cultures. We found that ROS inhibitors and caspase-1 inhibitors reduced PM2.5-induced neuronal injury. The results indicated that ROS played a key role in the PM2.5-induced IL-1β production and NLRP3 inflammasome activation. Furthermore, ROS and NLRP3 inflammasome activation was required for PM2.5-induced neuronal injury in neurons-microglia co-cultures.

## Conclusions

Taken together, in this study, we have shown that PM2.5 exposure aggravated oAβ-induced inflammation and neuronal injury in neurons-microglia co-cultures via increasing IL-1β production. Next, we demonstrated that PM2.5-induced IL-1β production in oAβ-stimulated microglia was possibly dependent on NLRP3 inflammasome activation. Using ROS inhibitors, we demonstrated that ROS was required for PM2.5-induced IL-1β production and NLRP3 inflammasome activation in oAβ-stimulated microglia. Lastly, we demonstrated that ROS and NLRP3 inflammasome activation was required for PM2.5-induced neuronal injury in neurons-microglia co-cultures. These data provide in vitro evidence that PM2.5 exposure increased neuronal injury and inflammation in microglia under AD context. Importantly, we showed that inhibition of NLRP3 inflammasome prevented PM2.5-induced neuronal injury and inflammation, suggesting that it might be a useful therapeutic target in alleviating the harmful health effects of PM2.5 exposure. Meanwhile, the findings should be further confirmed in animal models of AD.

## Abbreviations

AD: Alzheimer’s disease
Aβ: Amyloid beta
IL-1ra: IL-1 receptor antagonist
IL-1β: Interleukin 1 beta
NLRP3: NLR family pyrin domain containing 3
oAβ: Oligomeric amyloid beta
PM2.5: Fine particulate matter
ROS: Reactive oxygen species
